# A Culturally Tailored Mobile Health Intervention to Improve Quality of Life in Black Survivors With Prostate Cancer: Protocol for a Stratified Randomized Controlled Trial

**DOI:** 10.2196/81503

**Published:** 2026-03-24

**Authors:** Gaurav Kumar, Parisa Ghasemi, Adam C Alexander, Kathleen Dwyer, Jordan M Neil, Perry Cole, Patrick Beckford, Donald Reese, Everett Montgomery, Roland Odeleye, Mark A Harris, Yan D Zhao, Zsolt Nagykaldi, Andrew G McIntosh, Ernie Kaninjing, Mary E Young, Sabrina Dickey, Daniel J Morton, Opeyemi Bolajoko, Folakemi T Odedina, Lourdes G Planas, Michael S Businelle, Darla E Kendzor, Motolani E Ogunsanya

**Affiliations:** 1 TSET Health Promotion Research Center University of Oklahoma Health Campus Oklahoma City, OK United States; 2 Department of Family and Preventive Medicine University of Oklahoma Health Campus Oklahoma City, OK United States; 3 Fran and Earl Ziegler College of Nursing University of Oklahoma Health Campus Oklahoma City, OK United States; 4 The Multidisciplinary Health Outcomes Research and Economics (MORE) Lab Community Advisory Board University of Oklahoma Health Campus Oklahoma City, OK United States; 5 Department of Biostatistics and Epidemiology Hudson College of Public Health University of Oklahoma Health Campus Oklahoma City, OK United States; 6 Department of Urology Stephenson Cancer Center University of Oklahoma Health Campus Oklahoma City, OK United States; 7 School of Health and Human Performance Georgia College and State University Milledgeville, GA United States; 8 Mayo Foundation for Medical Education and Research Jacksonville, FL United States; 9 College of Nursing Florida State University Tallahassee, FL United States; 10 Department of Pediatrics University of Oklahoma Health Campus Oklahoma City, OK United States; 11 Stephenson Cancer Center University of Oklahoma Health Campus Oklahoma City, OK United States; 12 Mayo Clinic Comprehensive Cancer Center Jacksonville, FL United States; 13 College of Pharmacy University of Oklahoma Health Campus Oklahoma City, OK United States

**Keywords:** Black men, prostate cancer, cancer survivorship, symptoms monitoring, quality of life, mobile health, culturally-tailored interventions

## Abstract

**Background:**

Prostate cancer (CaP) disproportionately affects Black men in the United States, leading to significant disparities in incidence, survival, and quality of life (QoL). Treatment-related side effects, including urinary dysfunction, pain, fatigue, and psychological distress, contribute to poor long-term outcomes. There is an urgent need for culturally-tailored, technology-based interventions to support symptom self-management and survivorship care.

**Objective:**

This study aims to develop, refine, and evaluate the Survivorship App for Ethnically Diverse Black Prostate Cancer Survivors (SAFE-CaPS), a tailored mobile health intervention designed to enhance QoL, improve symptom self-management, and provide psychosocial support.

**Methods:**

This 12-month stratified randomized clinical trial will enroll 248 Black survivors with CaP (including native born, African-born, and Caribbean-born men) into the SAFE-CaPS intervention arm and standard care (SC) control group (1:1 allocation). Eligible participants are self-identified Black men (including native born, African-born, and Caribbean-born) aged 40 to 80 years with a confirmed CaP diagnosis within the past 5 years who own or are willing to use a smartphone. SC will consist solely of routine oncology or primary care follow-up, including clinic visits, referrals, and supportive services as needed, with no digital components. The SAFE-CaPS intervention, developed using the Insight platform, includes daily and weekly ecological momentary assessments of pain, urinary and bowel symptoms, sleep, fatigue, sexual function, mood, physical activity, and diet; adaptive educational modules addressing treatment-related side effects and culturally grounded survivorship concerns; automated symptom alerts prompting nurse follow-up and provider notification; and engagement features such as reminders, goal-setting, and literacy-appropriate content. Intervention content incorporates culturally tailored messaging, patient narratives, and feedback from prior qualitative work with ethnically diverse Black CaP. Stratified randomization will ensure balanced representation of ethnic subgroups. Primary outcomes include overall QoL. Secondary outcomes include specific symptom domains, mental health (depression and anxiety), health care engagement, and app acceptability. Assessments will occur at baseline, 3, 6, 9, and 12 months, and linear mixed models with intent-to-treat principles will evaluate intervention effects over time.

**Results:**

The study was funded in 2024, institutional review board approval was received in mid-2024, and the study began recruitment on August 24, 2025. Overall, 6 participants have been enrolled, with 2 in the SAFE-CaPS intervention and 4 in SC. Recruitment is expected to conclude in July 2027. Data analysis will occur in late 2027, with article preparation and publication planned for mid-2028 and early 2029. We hypothesize that SAFE CaPS participants will experience greater improvements in QoL, reduced symptom burden, and higher engagement in care compared to SC.

**Conclusions:**

This study will provide critical evidence on the feasibility, acceptability, and preliminary effectiveness of a culturally tailored mobile health intervention designed to enhance survivorship outcomes among Black survivors with CaP, informing future large-scale trials and real-world implementation.

**Trial Registration:**

ClinicalTrials.gov NCT06651359; https://clinicaltrials.gov/study/NCT06651359

**International Registered Report Identifier (IRRID):**

PRR1-10.2196/81503

## Introduction

Prostate cancer (CaP) remains a leading cancer diagnosis among men in the United States, with 313,780 new cases and 35,770 deaths projected in 2025 [1]. Among racial groups, Black men have the highest incidence rates nationwide at 191.5 cases per 100,000 compared to 114.5 per 100,000 among white men, and a higher CaP mortality rate (37.2 vs 18.1 deaths per 100,000, respectively) [2]. Black men have a greater likelihood of more advanced or aggressive disease at diagnosis, and they are frequently diagnosed at later disease stages, which limits treatment options and contributes to higher morbidity and mortality rates [3,4].

Disparities in CaP survival among Black men are influenced by genetic susceptibility, limited access to health care, family history, socioeconomic factors, and delayed detection [5,6]. Despite these disparities, the overall 5-year survival rate for Black men with CaP is high at approximately 97% [2]. However, survival does not equate to optimal posttreatment outcomes, as many Black men experience a disproportionate burden of treatment-related complications, psychosocial distress, and lower quality of life (QoL) [4,7-9]. CaP survivorship presents unique and persistent challenges, particularly for Black men, who often face disproportionate burdens of physical, emotional, and social distress [5,10]. Long-term treatment-related side effects such as sexual dysfunction, bowel and urinary incontinence, and mobility impairments can significantly diminish QoL [11-14]. Additionally, concerns about cancer recurrence, medical mistrust, and limited access to survivorship resources create further barriers to effective coping and reintegration into daily life [15-17]. These issues are often intensified by stigma surrounding sexual dysfunction, cultural beliefs about masculinity, and discomfort discussing intimate symptoms, which can delay help-seeking among Black men [9]. Addressing these multifaceted survivorship needs requires culturally tailored interventions that improve symptom management and psychosocial support. While many survivors with CaP face physical and emotional challenges, Black men often experience lower levels of functioning in these areas compared to White men [18-20]. Studies report slower recovery rates, decreased satisfaction with recovery, and higher levels of regret following prostatectomy among Black men [3,21,22].

The Black population in the United States is increasingly ethnically diverse, encompassing native-born Black men, African-born Black men, and Caribbean-born Black men [23,24]. While these groups share genetic similarities, their cultural beliefs, health care–seeking behaviors, and survivorship experiences vary significantly [25,26]. Across the African diaspora, Black men are at particularly high risk for CaP, but this risk is not uniform: native-born Black men have higher CaP mortality than African-born Black men and Caribbean-born Black men living in the United States, whereas some Caribbean-born Black men populations show an even greater likelihood of aggressive disease [27]. These patterns are likely driven by a combination of biological, environmental, behavioral, dietary, and geographic factors, including differences in risk exposures, screening practices, and access to high-quality care [4,28]. Studies suggest that perceptions of CaP, symptom management strategies, and engagement with health care differ based on cultural background, highlighting the need for culturally tailored research and interventions to address CaP disparities [8,9,29]. These needs also vary by ethnicity, particularly in areas such as communication about sexual health, stigma surrounding symptom disclosure, preferences for family involvement, and expectations of provider support, further reinforcing the need for tailored survivorship strategies [8,9,30]. Despite the high survivorship burden, Black men often report slower recovery rates, lower satisfaction with treatment outcomes, and higher levels of regret following prostatectomy compared to White men [18,20,31-33]. At the same time, many face systemic barriers to posttreatment support, including financial constraints, geographic challenges, and medical mistrust. These barriers may limit participation in traditional in-person survivorship programs, underscoring the need for scalable, culturally responsive approaches that address ethnic variation in survivorship priorities [3,34].

Mobile health (mHealth) interventions have shown great promise for supporting survivors with cancer by providing easy access to information, symptom tracking, and virtual counseling [35-39]. This technology is highly relevant for Black men with CaP, as more than 85% of Black Americans own smartphones [40], allowing them to access health resources remotely and overcome traditional barriers to care [41,42]. mHealth solutions also provide a convenient and scalable way to deliver tailored survivorship support, reducing reliance on in-person visits, which may be infrequent, inaccessible, or underused among Black men [39]. Additionally, these tools can deliver real-time symptom tracking, personalized resources, and interactive peer support, which have been shown to improve health outcomes in other cancer populations [39,43]. However, there are very limited existing mHealth interventions focusing on CaP [39,44], and none that are specifically designed for Black men or integrate culturally responsive features to enhance engagement and accessibility [39,44-46]. Given the high smartphone adoption rate among Black men and the potential for digital health interventions to reduce disparities, there is an urgent need to develop a culturally tailored mHealth solution that enhances QoL and addresses the unique survivorship challenges faced by Black survivors with CaP.

Given the persistent symptom burden, cultural variation in survivorship experiences, and limited engagement with supportive care among ethnically diverse Black men with prostate cancer, there is a need for theoretical frameworks that can both inform clinical targets and guide user engagement with digital resources. The Revised Wilson and Cleary Model (WCM) of health-related quality of life [47,48] provides a clinically grounded pathway linking biological function, symptom experience, functional status, and overall QoL, offering a clear rationale for monitoring and managing treatment-related side effects that disproportionately affect Black survivors with CaP. Complementing this approach, the Unified Theory of Acceptance and Use of Technology (UTAUT) [49,50] addresses factors that influence technology adoption and sustained use, including usability, cultural relevance, trust, social influence, and access to supportive conditions. Together, these frameworks provide a comprehensive foundation for designing interventions that are both clinically meaningful and acceptable to diverse Black survivor populations with CaP.

Therefore, this study aims to develop, culturally tailor, and evaluate the Survivorship App for Ethnically Diverse Black Prostate Cancer Survivors (SAFE-CaPS), a mHealth intervention designed to improve QoL, enhance symptom self-management, and strengthen psychosocial support among native-born, African-born, and Caribbean-born Black survivors with CaP. Specifically, this protocol describes the design and methodology of a stratified randomized clinical trial comparing SAFE-CaPS to standard care (SC). We hypothesize that participants receiving SAFE-CaPS will experience greater improvements in overall QoL, reductions in treatment-related symptom burden, and higher engagement in survivorship care compared with those receiving SC.

## Methods

### Study Design

This 12-month, 2-arm, stratified randomized clinical trial will use purposive sampling. A total of 248 Black survivors with CaP will be allocated in a 1:1 ratio to one of two study arms: (1) The SAFE-CaPS arm (n=124) where participants will receive access to the SAFE-CaPS app in addition to SC; or (2) Control arm (n=124) where participants will receive SC as recommended by their oncologist or other health care provider. SC will vary across participants and may include provider visits, referrals to specialists as indicated, and opportunities to receive support as needed. A stratified randomization approach will ensure a balanced representation of participants across diverse ethnic backgrounds. This study was reviewed and funded by the Department of Defense Prostate Cancer Research Program under Award Number HT942525PCRPIDA (Multimedia Appendix 1). This protocol was developed in accordance with the SPIRIT (Standard Protocol Items: Recommendations for Interventional Trials) 2025 guideline [51]. The completed SPIRIT checklist is included in Multimedia Appendix 2.

### Inclusion and Exclusion Criteria

Participants will be eligible if they (1) self-identify as Black, including native-born Black men, Caribbean-born Black men, and African-born Black men; (2) have a self-reported confirmed diagnosis of CaP within the last 5 years; (3) are between the ages of 40 and 80; and (4) own or will use a smartphone (Android or iOS).

Participants will be excluded if they do not meet the eligibility criteria listed above or if they are (1) unable or unwilling to provide a valid Social Security Number or Individual Taxpayer Identification Number, or do not meet U.S. residency requirements needed to comply with university reporting and compensation policies; or (2) currently enrolled in another structured CaP survivorship or support program.

### Study Recruitment and Procedure

Using purposive and snowball sampling, participants will be recruited through multiple channels across the United States via our current registries (more than 250 registered survivors with CaP), community-based approaches including our Community Advisory Board [52] members [30], fraternities, and the Inclusive Cancer Care Research Equity [48] for Black Men Consortium [53] and other existing research networks and clinics. The goal is to enroll a diverse population of Black survivors with CaP who meet the study criteria.

The first recruitment strategy will involve active collaboration with 1 of the co-investigators (medical oncologist), allowing recruitment through the urologic cancer clinics at the OU Health Stephenson Cancer Center (SCC). Clinical staff will identify eligible patients and assist with data collection during clinic visits. The second recruitment strategy will leverage the Clinical Research Data Warehouse at the SCC, enabling targeted recruitment. An electronic medical record data pull will identify potentially eligible participants. Identified participants will be contacted via mail and or phone. The third strategy will use existing research networks in Florida and Georgia, as well as a database of 108 Black survivors with CaP maintained by the senior author (MO). National social media recruitment will be conducted via ResearchMatch [54], a national health volunteer registry, in collaboration with an in-house marketing team to promote study participation through digital channels. Finally, community-based recruitment will be implemented through partnerships with the SCC Community Outreach and Engagement Core, the SCC African American Cancer Research CAB, and established community relationships, including faith-based organizations, the Prostate Cancer Survivorship CAB [30], the Inclusive Cancer Care Research Equity for Black Men Consortium [53], and the Mayo Clinic Minority Health & Health Equity Research Support initiatives [55]. Community education events will serve as engagement opportunities to enhance recruitment. A 1-page QR code-embedded recruitment flyer containing study details, eligibility criteria, and the study phone number will be distributed at community organizations, churches, and barbershops frequented by Black men. The multipronged recruitment strategy outlined has been successfully used in Black communities and is designed to address potential reluctance to participate in clinical trials [56,57]. Interested individuals will complete an online screening form or call the study phone number.

Participants in the SAFE-CaPS intervention group who do not own a smartphone will be provided with an Android smartphone and basic cell phone service to ensure equitable access. Those who already own an Android or Apple smartphone will use their personal device. All participants randomized to the intervention will receive a unique, single-use code to download the Insight app developed by the SCC mHealth Technology Shared Resource [58]. Upon entering the code, they will complete the baseline assessment and have access to the app for a 12-month period. Participants will receive practical instructions on using the app from the research team and there will be an option for a video tutorial outlining the features, and a video tutorial outlining the app's features will be available during the initial visit. This tutorial video can be saved to the phone's home screen for future reference, if needed by the participants. Participants will also be instructed on how to exclude the Insight app from “Do Not Disturb” mode settings on Android and Apple devices to ensure that ecological momentary assessments (EMAs) are received and completed in a timely manner. This guidance aims to reduce missed notifications due to phone settings and enhance EMA completion rates. The Insight app will serve as the platform for assessment and intervention for study groups. During the baseline assessment, participants will receive instructions on app features and complete prompted EMAs. These instructions are also available on demand via the “App Instructions” button on the app's home screen. Participants will complete a weekly, app-prompted EMA throughout the study. The app will also prompt participants to complete follow-up surveys at months 3,6, 9, and 12. Additionally, a subset of participants in the intervention group (n=30) will be invited to schedule an exit qualitative interview with a research team member to gather feedback on their experiences using the app.

**Figure 1:**
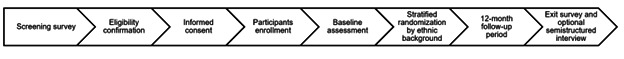
Participant flow for the Survivorship App for Ethnically Diverse Black Prostate Cancer Survivors stratified randomized clinical trial.

Participants in the control arm will receive SC as directed by their oncology providers, which may include clinic visits, specialist referrals, or support services. No additional educational materials, digital tools, or support will be provided by the study team beyond the scheduled assessments. They will not have access to the Insight app or EMAs. Participants in the control group will complete baseline and follow-up surveys through a secure REDCap (Research Electronic Data Capture; Vanderbilt University) link [59]. QoL assessments will be completed via REDCap at months 3, 6, 9, and 12. The planned flow of participants through screening, enrollment, randomization, intervention, and follow-up assessments is presented in Figure 1.

### Study Randomization

This study will use a 1:1 stratified randomized controlled trial design and will enroll 248 participants who will be randomly assigned to either the SAFE-CaPS intervention group (n=124) or the control group (n=124) using REDCap’s stratified randomization module. Stratification will be based on ethnic background to ensure a balanced representation across study arms: native-born Black men (n=100), African-born Black men (n=74), and Caribbean-born Black men (n=74). Within each subgroup, participants will be randomized in equal numbers to the intervention and control arms (native-born Black men: 50/50; African-born Black men: 37/37; Caribbean-born Black men: 37/37). This stratified approach ensures balanced subgroup representation and supports exploratory analyses assessing potential differences in survivorship experiences and intervention response across ethnic backgrounds.

Due to the nature of the intervention, blinding of participants, care providers, and recruitment staff is not feasible. However, the data analyst will be blinded to group allocation to reduce analysis bias.

### Mobile Health Platform

**Figure 2:**
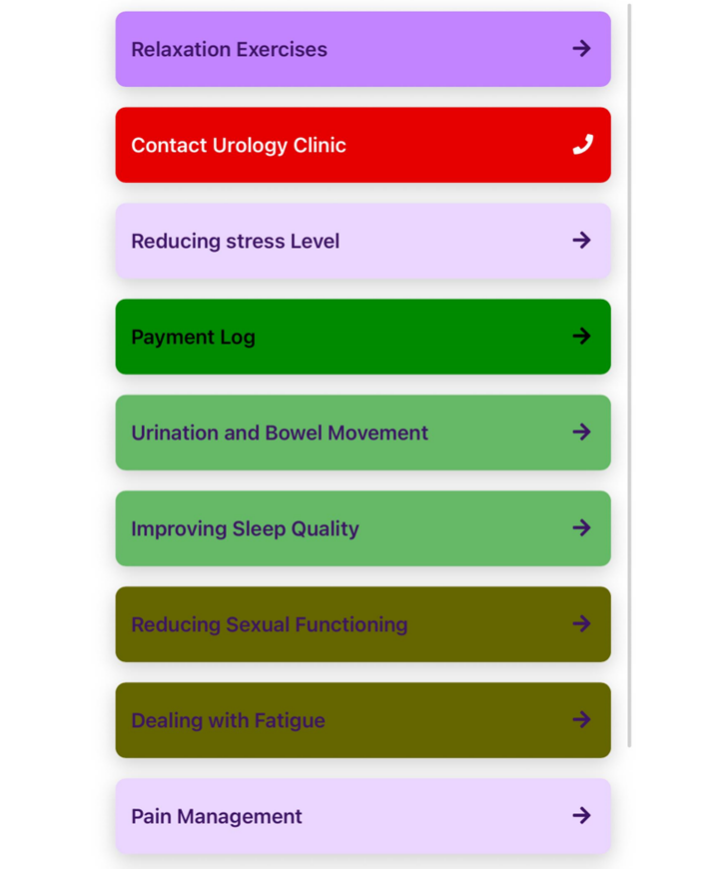
Home page of the Survivorship App for Ethnically Diverse Black Prostate Cancer Survivors (SAFE-CaPS) app.

The Insight mHealth Platform [58], developed by the SCC mHealth Technology Shared Resource [60], serves as the foundation for the SAFE-CaPS intervention. This state-of-the-art platform supports the rapid design, testing, and implementation of digital health studies, including secure EMAs, adaptive learning modules, automated symptom alerts, and provider notification tools, all within a HIPAA (Health Insurance Portability and Accountability Act)-compliant, encrypted infrastructure. EMA data are stored locally on study smartphones in a sandboxed, encrypted database and securely transmitted via encrypted channels to enterprise-grade Microsoft Azure servers, ensuring data confidentiality, integrity, and protection against unauthorized access. The platform has supported a broad portfolio of National Institutes of Health–funded clinical trials across cancer care, tobacco cessation, HIV prevention, chronic pain, and behavioral health [61-65], including studies specifically tailored for racial and ethnically diverse adults [61,66,67]. This evidence base demonstrates the platform’s scalability, cultural adaptability, and suitability for populations experiencing health inequities. Insight was selected for SAFE-CaPS because it provides oncology-specific safety features (eg, distress monitoring and nurse alerts), literacy-appropriate content options, and flexible cultural adaptation capabilities. The research team has collaborated closely with the mHealth Technology Shared Resource to develop and refine the SAFE-CaPS content and usability (Figure 2), ensuring alignment with study goals and adherence to clinical and ethical standards.

### Conceptual Frameworks

The SAFE-CaPS study is guided by an adaptation of the Revised WCM of QoL [47,48], which was informed by findings from our extensive integrative review [8] and prior qualitative studies [9,29,68]. The Revised WCM model provides a structured approach for understanding the impact of biological function, symptom experience, functional status, and general health perceptions on QoL among Black men with CaP [48]. The model posits that biological function (eg, CaP-related pathophysiology) influences symptom status (eg, urinary dysfunction, fatigue, and depression), which in turn affects functional status (eg, ability to perform daily activities) [48,69]. These factors shape an individual’s general health perception, ultimately determining their overall QoL, as measured by the Functional Assessment of Cancer Therapy-Prostate (FACT-P) questionnaire. The SAFE-CaPS intervention leverages this framework to enhance symptom monitoring, self-management, and early intervention to improve functional status and QoL outcomes.

**Figure 3:**
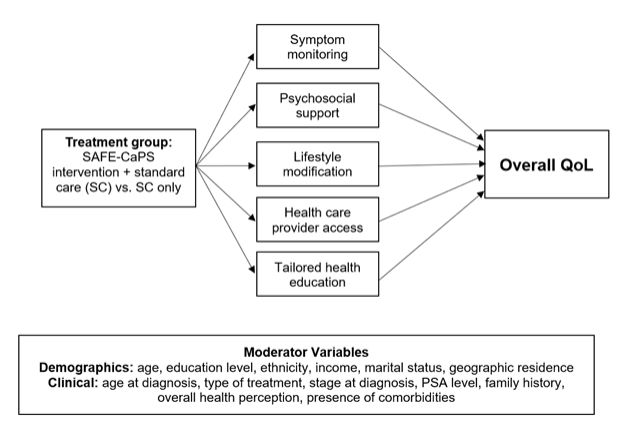
Proposed model of treatment mechanism. PSA: prostate-specific antigen; QoL: quality of life; SAFE-CaPS: Survivorship App for Ethnically Diverse Black Prostate Cancer Survivors.

Demographic factors (eg, age, education, ethnicity, income, marital status, and geographic residence) and clinical variables (eg, age at diagnosis, treatment type, disease stage, prostate-specific antigen level, family history, overall health perception, and comorbidities) may moderate the impact of the intervention on QoL. The integration of the WCM framework within the SAFE-CaPS intervention is designed to account for individual differences, ensuring a comprehensive, culturally tailored approach to improve QoL among Black survivors with CaP (Figure 3).

**Figure 4:**
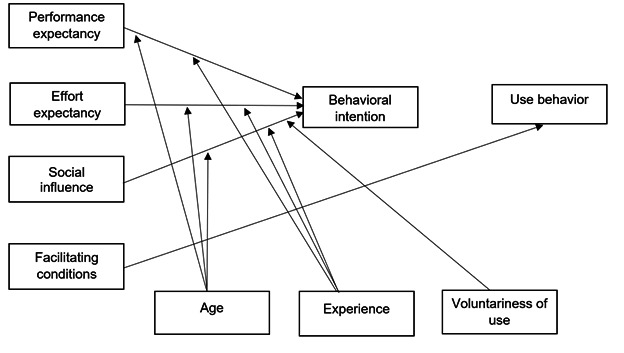
Proposed Unified Theory of Acceptance and Use of Technology model.

Additionally, the study incorporates the UTAUT framework to assess participant engagement and technology adoption [49]. The UTAUT model evaluates 4 key constructs that have been shown to influence behavioral intent and sustained app use (Figure 4) [49,70]. Performance expectancy refers to whether participants believe the intervention app will help them manage symptoms and improve their health behaviors. Effort expectancy captures perceptions of ease of use and overall accessibility. Social influence considers the extent to which health care providers, caregivers, and peers encourage engagement with the app. Facilitating conditions assess the availability of necessary resources and technical support to enable effective use of the intervention. Additionally, age serves as a moderating variable in the UTAUT model [49,70]. By integrating WCM for health outcomes and UTAUT for technology adoption, the study ensures a comprehensive evaluation of intervention effectiveness, allowing researchers to measure both clinical improvements in survivorship and behavioral factors affecting participant engagement. This dual-framework approach will inform future enhancements and scalability of the SAFE-CaPS intervention.

### Intervention (SAFE-CaPS)

The SAFE-CaPS intervention is designed to provide real-time symptom monitoring and management of CaP-related symptoms while integrating culturally relevant content and psychosocial support. Acknowledging the importance of emotional well-being, the intervention includes resources for mental health support to improve coping with psychological challenges related to CaP survivorship.

The development of the SAFE-CaPS app was guided by a Community-Based Participatory Research approach, ensuring that the needs and lived experiences of Black men with CaP shaped its design and implementation [8,9,29,30]. The intervention is built upon findings from our prior work exploring QoL among ethnically diverse Black men with CaP [8,9,29]. This research identified key survivorship concerns, including symptom burden, psychosocial distress, and unmet informational needs, highlighting the necessity of a culturally responsive intervention. To increase relevance and usability, the app underwent iterative refinement in collaboration with a CAB composed of ethnically diverse Black men survivors with CaP [30]. Through focus groups, usability testing, and feedback from Black men experiencing CaP survivorship, the app was tailored to reflect their preferences, values, and health care experiences. Unique features include culturally relevant health education and an emphasis on peer support and interactions with trusted health care providers. Additionally, the intervention addresses historical health care mistrust by integrating educational modules that empower users to navigate the health care system and communicate effectively with providers.

A key feature of the SAFE-CaPS app is its incorporation of culturally tailored content, recognizing potential within-group differences among Black men from native-born, African-born, and Caribbean-born backgrounds. The intervention also acknowledges historical mistrust in health care and includes educational modules to enhance users' understanding of the health care system and encourage effective communication with providers. The intervention provides both dynamic and static resources, featuring content that adapts to user inputs and a comprehensive content library. The intervention also enables participants to log and share their symptoms with health care providers or designated proxies, supporting timely symptom reporting. Furthermore, the intervention uses behavioral engagement strategies, including push notifications and reminders, to encourage consistent participation. Using a longitudinal tracking approach, the SAFE-CaPS intervention captures detailed insights into the survivorship journey, including symptom burden, QoL, health behaviors (eg, physical activity, diet, and sleep), app usage patterns, and user-reported outcomes through weekly EMAs and quarterly follow-up surveys.

**Figure 5:**
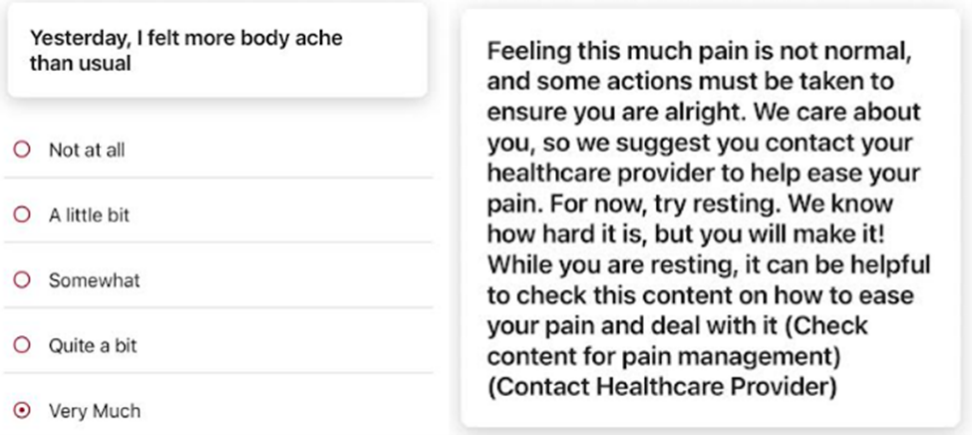
Example ecological momentary assessment item from risk assessment (left) and adaptive educational material (right).

SAFE-CaPS includes daily assessments of key symptoms, including pain, sleep disturbances, fatigue, weight gain, urinary frequency and difficulty, bowel control issues, depression, anxiety, and worry. Participants also have the option to add additional comments in an open-text section to report any concerns or symptoms not captured in the standard items. Specific symptoms will trigger alerts based on frequency or distress levels, notifying both the study team and the designated health care providers selected by participants (Figure 5).

**Figure 6:**
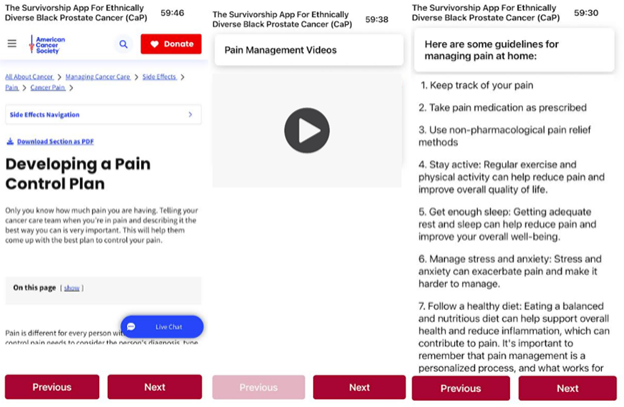
Samples of educational content.

These alerts will prompt adaptive educational materials that provide self-management strategies until medical consultation is obtained (Figure 6). Specific alert triggers include urinary urgency, difficulty urinating, hematuria, constipation, blood in stool, pain, worry, or depression. Through these integrated components, SAFE-CaPS delivers a holistic and culturally responsive approach (developed with input from Black survivors with CaP and community advisors to ensure that language, imagery, and messaging reflect the lived experiences, preferences, and cultural values) to support Black men in navigating the complexities of CaP survivorship.

### Control Group (SC)

Participants in the control group will not have access to the SAFE-CaPS intervention content. Still, they will receive standard survivorship care, including routine medical follow-ups and referrals as needed through their health care providers.

### EMA

The SAFE-CaPS daily EMAs will include brief, real-time assessments of symptom status, functioning, and overall QoL. The goal is to minimize participant burden while capturing the essential data points necessary for the study’s analyses.

### Data Collection

Assessments will be conducted at baseline, post intervention, and interim time points at 3, 6, and 9 months. The assessment schedule is outlined in Table 1 (details of the assessment measures are provided in the Multimedia Appendix 3).

**Table 1:** Overview of the questionnaire assessments.

EMA^a^ measures		Baseline (week 1)	Assessments (week 1-12)	Follow-up
**Weekly^b^ assessments (domains)**				
	Sociodemographic characteristics	✓		
	Clinical characteristics	✓		
	The positive and negative affect schedule	✓	✓	✓
**Daily assessments (domains)**				
	Pain level and impact	✓	✓	
	Sleep quality	✓	✓	
	Sexual functioning	✓	✓	
	Fatigue level	✓	✓	
	Weight gain	✓	✓	
	Urine frequency and difficulty	✓	✓	
	Bowel control	✓	✓	
	Physical activity frequency	✓	✓	
	Dietary records	✓	✓	
**Monthly^c^ assessments**				
	PHQ-4^d^	✓		✓
	FACT-P^e^	✓		✓

^a^EMA: ecological momentary assessment.

^b^For weekly assessments, the follow-up was done for months 6-12.

^c^For monthly assessments, the follow-up was done for months 3-12.

^d^PHQ-4: Patient Health Questionaire-4.

^e^FACT-P: Functional Assessment Cancer Therapy–Prostate.

### One-Time Assessments

At baseline, sociodemographic and clinical characteristics will be collected, including age, education level, ethnicity, marital status, geographic residence, and clinical history (eg, age at diagnosis, stage of cancer, treatment type, family history of CaP, treatment status, prostate-specific antigen level, and comorbidities).

#### Weekly EMAs

Mood will be assessed at baseline and weekly for the first 12 weeks and during the week following each QoL assessment at 3, 6, 9, and 12 months, totaling 15 weeks of evaluation. The Positive and Negative Affect Schedule will be used to measure affect, consisting of two 10-item scales assessing positive and negative mood [71]. Participants will rate the intensity of several dimensions of their affect on a 5-point scale (1=very slightly or not at all; 5=very much), with higher scores indicating greater positive or negative affect [71].

#### Daily EMAs

The SAFE-CaPS group will have access to the app-based intervention for 12 weeks. During this time, participants will complete daily assessments of various symptoms, including pain intensity and impact, sleep quality, fatigue levels, weight changes, urinary frequency and difficulty, body aches, bowel control, sexual function, and depression or anxiety. Additional measures will assess physical activity frequency, duration, and dietary intake (fruit and vegetable consumption). Participants will also complete daily EMAs for 1 week following their 6, 9, and 12-month QoL assessments. Each daily evaluation is expected to take approximately 5–10 minutes to complete.

#### Quarterly Assessments

QoL and mental health outcomes, including depression and generalized anxiety disorder, will be evaluated at baseline and 3, 6, 9, and 12 months. These assessments are designed to take approximately 5 minutes to complete.

#### Post Intervention Survey

Twelve months post-randomization, participants assigned to SAFE-CaPS will complete a structured online survey via REDCap to assess intervention engagement, usability, and changes in health behavior, based on the UTAUT framework. The survey will evaluate participants' responses to the 4 key intervention constructs: performance expectancy, effort expectancy, social influence, and facilitating conditions, along with their final behavioral intent.

#### Exit Interview

A subset of 30 intervention participants will be invited to participate in semistructured interviews and focus groups conducted via Zoom to collect qualitative data on the usability, engagement, and perceived benefits of the SAFE-CaPS app. The interview guide will explore several key domains related to participants’ experiences with the intervention. Specifically, the interviews will delve into their CaP survivorship journey, including interactions with health care providers, the long-term impact of treatment, and motivations for participating in the study. Questions will assess participants’ affective attitude (ie, feelings about the intervention), perceived effectiveness (ie, perceived improvements in health and well-being), and self-efficacy (ie, confidence in carrying out intervention-related behaviors). The domain of burden will capture perceived effort, challenges, and time investment associated with the intervention. Intervention coherence will assess how well participants understand the purpose and functioning of the app. Additionally, participants will be invited to offer recommendations for improvement, describe their ideal model of survivorship support, and share whether they would recommend the app to others facing similar experiences.

### Sample Size and Power Analysis

The sample size required to detect a significant change in the primary outcome (FACT-P Total Score) was estimated using a 2-sample *t* test. Although FACT-P will be assessed at baseline and at 3, 6, 9, and 12 months, the sample size calculation was based on detecting a statistically significant difference between groups at the 12-month timepoint, which serves as the primary endpoint. The calculation assumed a significance level (α) of 0.05, 80% power, and a 1:1 randomization ratio. Based on prior research by Cella et al [72], which reported an effect size between 0.39 and 0.58, the sample size was conservatively determined using the smaller effect size (0.39). Under these assumptions, a minimum of 105 participants per group is required to detect a meaningful difference in FACT-P scores.

We anticipate that 15% of the full sample will be lost to attrition; therefore, the final target sample size was set at 124 participants per group (N=248 total). A participant sampling plan will be implemented to ensure adequate representation of ethnic groups and balanced allocation across intervention and control conditions, in alignment with the randomized controlled trial design.

### Measures

This study will use a range of validated instruments and self-reported measures to assess key constructs related to QoL, symptom burden, psychosocial well-being, lifestyle behaviors, and health care access among Black men with CaP.

#### Primary Outcome

The primary outcome will be QoL, which will be assessed using the (FACT-P) questionnaire [72] at baseline, 3, 6, 9, and 12 months. FACT-P is a 39-item instrument that evaluates physical, emotional, and functional well-being, as well as CaP-specific concerns such as weight loss, appetite, pain, urinary symptoms, bowel function, and sexual health. Scores will be transformed onto a 0-100 scale, with higher scores indicating better QoL.

#### Secondary Outcomes

App engagement and usability will be evaluated based on frequency and duration of use, symptom-tracking interaction, and engagement with educational content. Technology acceptance and behavioral intent will also be assessed using the UTAUT framework, which evaluates performance expectancy, effort expectancy, social influence, and facilitating conditions. These outcome variables will be analyzed at multiple time points to determine the effectiveness of the SAFE-CaPS intervention in enhancing QoL, improving mental health, and supporting symptom self-management among Black survivors with CaP. Additionally, semistructured interviews and focus groups will be conducted to explore app usability, engagement, and perceived benefits.

#### Analytic Plan

The statistical analyses for the SAFE-CaPS study will include descriptive, bivariate, and multivariate methods to assess the intervention's impact on QoL, mental health, symptom management, and app engagement. Each variable will be examined for distribution, range, mode, median, and mean (SD). Skewness and kurtosis will be determined for all ratio-level variables, while frequency distributions will be calculated for nominal- and ordinal-level variables. Descriptive statistics will be used to summarize all study variables, and bivariate statistics will be computed to assess relationships among theoretical constructs.

Differences in demographic and clinical characteristics between the 2 groups will be analyzed using a 2-tailed independent *t* test, Fisher exact test, and the chi-square test. Intervention usage will be analyzed using descriptive and inferential statistics for the number of EMAs completed, alerts triggered, and views of the educational section. An adjusted linear regression mixed-effect model will assess the impact of treatment group assignment on FACT-P scores at 12 months, adjusting for relevant covariates such as age, socioeconomic status, treatment type, ethnicity, and cancer stage. Preliminary analyses will include checks for multicollinearity and score distributions to ensure model validity. The goal is to determine the effect of the intervention on QoL as measured by FACT-P, independent of these baseline characteristics.

Chi-square analyses and *t* tests will examine baseline differences between ethnic groups. One-way ANOVA and correlations will be conducted to determine whether demographic and clinical variables are significantly associated with perceived acceptability, accessibility, and engagement. Due to the preliminary nature of these analyses, only covariates significant at *P*<.05 will be included in the final models. Cronbach α will assess the reliability of the multi-item instrument to determine the internal consistency of the model used to measure the constructs of the proposed framework, as shown in Figure 3. A Cronbach α value ≥0.70 will be considered acceptable for internal consistency [73]. Data will be analyzed using SPSS (version 29; IBM Corp).

### Qualitative Analysis Plan

Qualitative data from postintervention interviews will be analyzed using a reflexive thematic analysis approach to explore participants’ experiences with the SAFE-CaPS mHealth app. All interviews will be transcribed verbatim by a professional transcription service with established safeguards to protect Protected Health Information, and transcripts will be coded using ATLAS.ti v24 (Lumivero/ATLAS.ti Scientific Software Development GmbH). Two researchers (MO and GK) will independently review transcripts, develop initial codes, and iteratively refine a shared codebook through discussion and consensus. Codes will then be organized into broader categories and themes that capture perceptions of usability, acceptability, cultural relevance, and factors influencing engagement with the intervention, with attention to variation across ethnic subgroups. Emerging themes will be reviewed collaboratively with the research team to ensure analytic rigor. Qualitative findings will be integrated with quantitative outcomes using an explanatory sequential mixed-methods approach, allowing qualitative insights to contextualize, explain, and complement observed quantitative patterns to provide a comprehensive understanding of intervention usability and impact.

### Study Compensation

Participants (N=248) will receive US $50 each for completing both the baseline and postintervention assessments, totaling US $100 per participant. In addition, all participants will receive US $25 for completing each interim survey at 3, 6, and 9 months (a total of US $75). Participants in the SAFE-CaPS arm who complete the semistructured exit interview (n=30) will receive an additional US $50 payment. To enhance EMA compliance, SAFE-CaPS participants (n=124) will receive US $2 per day for completing daily assessments once a day over 15 weeks (up to US $210) and US $20 per completed weekly assessment (up to US $300). Thus, participants in this arm may earn up to US $685, or US $735 if they also complete the exit interview. Participants in the control arm may earn up to US $175 in total. Compensation will be provided weekly via a reloadable study credit card [74]. Participants will not be compensated for on-demand app usage or engagement with intervention components. Although the total potential payment varies by arm, the additional amounts in SAFE-CaPS accurately reflect the increased time and effort required for intensive EMA data collection. This structure was reviewed and approved by the University of Oklahoma Health Campus Institutional Review Board (IRB). To guard against bias, total compensation received will be captured and evaluated as a covariate in sensitivity analyses.

### Ethical Considerations

The SAFE-CaPS study will adhere to strict ethical guidelines to protect participants’ rights, privacy, and safety. The study protocol has been approved by the University of Oklahoma Health Campus Institutional Review Board (OUHC IRB; protocol number 17116). This trial is registered at ClinicalTrials.gov (NCT06651359). All participants will provide written informed consent after being fully informed of the study purpose, procedures, risks, benefits, and voluntary nature of participation, including the right to withdraw at any time without penalty.

To protect privacy and confidentiality, all participant data will be deidentified, encrypted, and stored on secure, password-protected computers and servers. Audio-recorded interviews will be professionally transcribed, and all transcripts will be deidentified. Only authorized study personnel will have access to identifiable data. Additional protections, including user access controls, password authentication, auto-logout features, and audit trails, will be implemented to prevent unauthorized access to Protected Health Information. Participants who experience distress or report clinically concerning symptoms will be referred to appropriate health care providers, and the app’s automated alert system will notify the study team when severe symptoms are reported to facilitate timely follow-up. Ethical oversight will be maintained through ongoing IRB monitoring and adherence to all federal and institutional regulations governing human subjects’ research. Participants will receive monetary compensation for completing study assessments across the 12-month period, with payments distributed at each assessment point. Participants in the SAFE-CaPS arm may earn up to US $685, or up to US $735 if they complete the exit interview, whereas participants in the control arm may earn up to US $175 in total.

## Results

The SAFE-CaPS study received funding in January 2024, and IRB approval was received in May 2024. Study recruitment began on August 24, 2025. Overall, 6 participants have been enrolled, including 2 in the SAFE-CaPS intervention arm and 4 in the SC group. Recruitment is expected to conclude in July 2027. Data analysis is anticipated to occur toward the end of 2027, with article preparation and publication planned for mid-2028 and early 2029. The SAFE-CaPS study is expected to improve QoL, mental health, and symptom management among ethnically diverse Black men with CaP. It is anticipated that participants in the intervention group will demonstrate significant improvements in FACT-P scores, along with reductions in depression and anxiety symptoms over time. Real-time symptom monitoring is expected to enhance self-management and facilitate timely intervention, resulting in fewer severe symptom episodes and improved health outcomes. High app engagement is anticipated, with frequent use of symptom tracking, educational content, and provider alerts. Increased engagement may be associated with better health behaviors, including increased physical activity and improved dietary intake. Exploratory analyses may reveal differences in app adoption based on sociodemographic factors, enabling the identification of potential disparities in digital health use. Furthermore, qualitative findings are expected to provide insights into facilitators and barriers to app usability, informing future refinements of the intervention and guiding the scalability and adaptation of the SAFE-CaPS model for broader implementation.

## Discussion

### Principal Findings

The SAFE-CaPS study is expected to demonstrate that participants receiving the culturally tailored mHealth intervention will experience greater improvements in QoL, reductions in symptom burden, and higher engagement in survivorship care than those receiving SC. We anticipate that real-time symptom monitoring, adaptive educational support, and culturally grounded content will enhance symptom self-management, reduce distress, and facilitate more timely health care follow-up. Qualitative findings are also expected to illuminate user experiences, highlight facilitators and barriers to engagement, and guide iterative refinement of the app.

### Comparison With Prior Work

Prior research has consistently shown that Black men with CaP experience a higher symptom burden, lower engagement in survivorship care, reduced QoL, and significant disparities in health outcomes compared to other racial groups [3,8,39,75]. Digital health solutions have demonstrated promise in improving symptom management, mental health, and survivorship outcomes across various cancer populations [76]. However, existing CaP mHealth interventions have rarely been designed specifically for Black men, nor have they accounted for within-group cultural diversity among native-born, African-born, and Caribbean-born survivors with CaP. SAFE-CaPS advances this literature by integrating culturally relevant educational resources and survivorship priorities identified directly from diverse Black survivors with CaP and community advisors, addressing a critical gap in prior mHealth and survivorship studies.

### Strengths and Limitations

The SAFE-CaPS study has several key strengths that distinguish it as a pioneering mHealth intervention for Black survivors with CaP. First, its culturally tailored design enables the intervention content, symptom tracking, and psychosocial support features to directly address unique survivorship challenges faced by Black men, particularly considering historical health care mistrust and disparities in symptom management. Second, the app includes real-time symptom monitoring with a tiered alert system: when a participant reports a symptom that exceeds a prespecified frequency or distress threshold, an alert is routed to the research team, who will contact the participant within 24 hours and, with the participant’s permission, notify the designated health care provider for clinical follow-up. Third, the study uses a comprehensive evaluation framework that integrates quantitative assessments (FACT-P and Patient Health Questionnaire-4 scales) with qualitative data from semistructured interviews to assess the effectiveness, feasibility, engagement, and ongoing refinement of the app. Additionally, the use of validated theoretical frameworks (WCM and UTAUT) strengthens the study’s conceptual foundation, linking clinical outcomes, psychosocial factors, and technology adoption to enhance survivorship care.

Despite its strengths, the study has several limitations. One potential challenge is variability in intervention engagement, which may be influenced by factors such as digital literacy, socioeconomic status, and access to technology, potentially limiting the generalizability of the findings. To address this, participants will receive technical support and training to enhance the usability and engagement of the intervention. A further limitation is the potential for differential retention between study arms, as participants in the SAFE-CaPS group may have greater motivation to remain in the study due to access to the intervention and associated compensation, which could contribute to higher loss to follow-up in the usual care group. Retention will be monitored across study arms, and results will be interpreted with consideration of potential differential attrition. Another limitation is reliance on self-reported data for QoL and symptom tracking, which may introduce recall and response bias. However, the use of validated assessment instruments and objective app engagement metrics will enhance data reliability and mitigate some concerns. Despite these limitations, demonstrating the preliminary efficacy of the SAFE-CaPS app will provide valuable insights into the feasibility and effectiveness of culturally tailored digital interventions for improving CaP survivorship outcomes in Black men.

**Future Directions**

Findings from this study will guide the refinement of SAFE-CaPS, including enhancing usability features, tailoring educational content, and improving the symptom alert process. Future work may involve adapting the intervention for broader geographic implementation, testing the app in a larger multisite randomized clinical trial, and exploring integration with electronic health records for automated clinical alerts. The study team plans to disseminate findings through peer-reviewed publications, national conferences, community presentations, and partnerships with cancer centers and survivorship networks. Ultimately, SAFE-CaPS has the potential to inform scalable, equitable digital survivorship models for diverse and underserved cancer survivor populations.

### Conclusions

The SAFE-CaPS study represents a significant effort to address persistent survivorship disparities among Black prostate cancer survivors through a culturally tailored, mHealth–enabled approach. By combining real-time symptom surveillance, tailored education, and community-informed content, this intervention aims to provide a scalable and accessible tool to support improved survivorship outcomes. The results of this protocol-driven trial will contribute critical evidence to the fields of digital oncology, health equity, and mHealth implementation, and will inform future policy, clinical integration, and larger-scale trials aimed at expanding culturally responsive survivorship care.

## Appendix

Multimedia Appendix 1 Department of Defense peer-review summary report.

Multimedia Appendix 2 SPIRIT 2025 checklist.

Multimedia Appendix 3 Overview of scheduled assessment.

## Abbreviations

CaP: prostate cancer
EMA: ecological momentary assessment
FACT-P: Functional Assessment of Cancer Therapy – Prostate
HIPAA: Health Insurance Portability and Accountability Act
IRB: institutional review board
mHealth: mobile health
QoL: quality of life
REDCap: Research Electronic Data Capture
SAFE-CaPS: Survivorship App for Ethnically Diverse Black Prostate Cancer Survivors
SC: standard care
SCC: Stephenson Cancer Center
SPIRIT: Standard Protocol Items: Recommendations for Interventional Trials
UTAUT: Unified Theory of Acceptance and Use of Technology
WCM: Wilson and Cleary Model

## Data Availability

The datasets generated or analyzed during this study will be available from the corresponding author upon reasonable request.
