# Supplementary material for: A Culturally Tailored Mobile Health Intervention to Improve Quality of Life in Black Survivors With Prostate Cancer: Protocol for a Stratified Randomized Controlled Trial
Source: JMIR Res Protoc. 2026 Mar 24;15:e81503. doi: 10.2196/81503 (PMC13058526; doi:10.2196/81503)
Supplement: Multimedia Appendix 1 [file resprot_v15i1e81503_app1.pdf]

Department of Defense
U.S. Army Medical Research and Development Command
Congressionally Directed Medical Research Programs
Fiscal Year 2023 Prostate Cancer Research Program
Health Disparity Research Award - New Investigator- Clinical Trial
Peer Review Summary Statement

CDMRP Log Number: PC230551
Grants.gov ID Number: GRANT13963800
Review Panel: Health Disparity Research - 3
Discussion Period: 10/17/2023-10/18/2023

Project Duration: 36 months
Budget Requested: $1,303,321
Direct Costs: $898,842
Indirect Costs: $404,479

Title: Development and Preliminary Evaluation of a Tailored mHealth App Designed to Improve Quality-of-Life Outcomes in Prostate Cancer Survivors
Principal Investigator: Motolani Ogunsanya
Performing Organization: Oklahoma, University of, Health Sciences Center
Contracting Organization: Oklahoma, University of, Health Sciences Center

OVERVIEW

The Principal Investigator (PI) of this application proposes to develop a mobile health (mHealth) intervention to specifically address the needs of ethnically diverse Black prostate cancer survivors and test the hypothesis that the tailored mHealth app, Survivorship App For Ethnically Diverse Black Prostate Cancer Survivors (SAFE-CaPS), will lead to improved quality of life (QoL) outcomes among ethnically-diverse Black prostate cancer survivors. The project's specific aims are to (1) continue developing and refine an mHealth app for Black men with prostate cancer to address symptom-related and psychosocial support needs; (2) pilot test the mHealth app on overall prostate cancer QoL; and (3) assess the app engagement, accessibility, and acceptability. A small-scale, early randomized clinical trial will be performed. The intervention is the use of the SAFE-CaPS app by Black men with prostate cancer. The projected outcomes are enhanced survivorship experiences for the ethnically diverse Black prostate cancer survivors.

	**Average Score**	**Standard Deviation**
**Overall Evaluation**<br>*Rating Scale: 1.0 (highest merit) to 5.0 (lowest merit)*	1.8<br>**(Excellent)**	0.3
**Criteria**<br>*Rating Scale: 10 (highest merit) to 1 (lowest merit)*	**Average Score**	
**Impact**	8.6	
**Research Strategy and Feasibility**	7.0	
**Clinical Strategy**	7.3	
**Personnel**	8.5	
**Research Transition Plan**	8.5	

SCORED CRITERIA

Impact
Average Score: 8.6

Scientist Reviewer A

Strengths: The proposed research addresses the FY23 PCRP HDRA overarching challenges of improving QoL, advancing health equity, and reducing disparities in prostate cancer outcomes within an ethnically diverse population of Black men. Short-term outcomes of the project will include an understanding of which subgroups of Black men are at greatest risk of poor posttreatment QoL outcomes during prostate cancer survivorship, allowing for development of culturally tailored tools with which to intervene, in order to improve outcomes for those who are at greatest risk of experiencing poor QoL. By exclusively focusing on Black men from multiple ethnic backgrounds, the study has the ability to shed light on factors that contribute to health disparities in such groups, during a prostate cancer survivorship period. Long-term outcomes of the project will include earlier identification of men at greatest risk for poor QoL which could significantly improve QoL for Black men and reduce prostate cancer QoL health disparities.

Weaknesses: It is possible that the mHealth app will not be embraced by all subgroups of interest to the study. Continued efforts will be necessary to engage Black prostate cancer survivors who do not readily embrace the app. The use of the app may have a limited effect on QoL outcomes; examining the features and aspects of the app that are most useful to impacting QoL will be important. The study population that will be randomized is fairly small, and the pilot data generated are based on small numbers (n = 31). Therefore, the generalizability of the study findings may be limited in terms of applicability to Black male ethnic subgroups in the United States. The semiqualitative data will be very important for developing themes to understand how the mHealth app could ultimately be used more broadly, beyond the 3-year study and across Black male subgroups.

Scientist Reviewer B

Strengths: If successful, the implementation and efficacy of a smartphone app for symptom management and psychosocial support would improve survivorship for men with prostate cancer. The proposal serves to address the FY23 PCRP overarching challenge of improving QoL to enhance outcomes and overall health and wellness for those impacted by prostate cancer. The SAFE-CaPS health app may provide accessible guidance and a platform for accessing reliable information.

Weaknesses: It is not clear from this proposal whether a health app would be readily used in resource limited settings where diverse populations are treated.

Consumer Reviewer

Strengths: Prostate cancer affects Black men in the United States more than any other group, and survivors often face challenges that impact their QoL; therefore there is a crucial need to provide enhanced support tailored to their unique needs. Recent advancements in mobile health technology offer promising avenues for personalized care among cancer survivors. However, there currently exists no dedicated app specifically designed for Black prostate cancer survivors. This proposal suggests the development and testing of an accessible and user-friendly mobile health app named SAFE-CaPS. This study will be to evaluate how technology, in the form of the SAFE-CaPS, can improve the QoL for ethnically diverse Black prostate cancer survivors. The impact of the SAFE-CaPS app can contribute to the broader goal of eliminating disparities in Black men with prostate cancer. This proposed study highlights the importance of leveraging technology to bridge gaps in health care and promote well-being

among underserved populations, given the stigma observed among men who have survived prostate cancer. This technology has the potential to empower users with tailored strategies and knowledge to manage their health effectively and make informed decisions. This study will take a look at the stigma surrounding prostate cancer that arises because prostate cancer impacts traditional ideas of masculinity, involving reproductive organs and side effects like problems with incontinence, bowel control, sexual difficulties, and impotence. Additionally, this research will add study of Black immigrants, thereby contributing to narrowing disparities in prostate cancer mortality, especially important given the increasing number of Black immigrants within the United States.

Weaknesses: No weaknesses were noted.

Research Strategy and Feasibility
Average Score: 7.0

Scientist Reviewer A

The proposed study consists of a quasi-randomized clinical trial comparing a tailored app to standard of care to improve QoL among ethnically diverse Black men who are prostate cancer survivors. This work is expected to advance the understanding of the efficacy and implementation of a smartphone-based app for symptom management and psychosocial support in ethnically diverse Black men diagnosed with prostate cancer. Multiple metrics of the primary study end point, QoL, as well as complementary approaches to subject enrollment are proposed. It is expected that overall QoL will be higher for those assigned to use the app as compared to the control group. Techniques such as purposive and snowball sampling will be used to recruit participants from across the United States via their current registries (currently more than 250 registered prostate cancer survivors), community-based approaches (using community advisory board members, fraternities, Inclusive Cancer Care Research Equity (iCCaRE) for Black Men Consortium, other existing research networks, and clinics at the Stephenson Cancer Center and the Oklahoma City VA Medical Center. A conceptual framework based on the model of Wilson and Cleary is used to posit study associations.

Strengths: The study leverages findings from the PI's recently completed DOD-funded study, building a mobile health app to understand factors that can improve QoL among Black prostate cancer survivors from ethnically diverse backgrounds. The domains of factors selected that are expected to impact QoL are theory driven. The study offers novel and thoughtful strategies for improving QoL in a subset of prostate cancer survivors whose experiences are lesser known and understudied. The work has the potential to improve QoL and reduce disparities in underserved prostate cancer survivors.

Weaknesses: The study concepts are built on findings from a relatively small sample size of patients from funded pilot work completed by the PI. The current study will also consist of fairly modest sample sizes. It is not clear that baseline metrics are following a timeline that is comparable for all prostate cancer survivors enrolled in the study, in terms of time since completed treatment.

Scientist Reviewer B

Strengths: The investigators have noted that there is strong interest in app development; the PI identified 9 apps, only 1 of which remains in use, and few if any included culturally diverse populations. Previous funding has allowed for development of a prototype SAFE-CaPS app, and the specific aims look to refine the app and test its ability to improve QoL.

Weaknesses: The investigators state in Aim 1 that the goal is to develop and refine an mHealth app for Black men with prostate cancer. In Aim 2, they pilot test the app and use randomized men to test whether

the app significantly improves symptom management and overall better QoL. During the grant time frame, it is unclear whether this is feasible. The app requires testing of effectiveness, efficiency, and usability, as the investigators note, and the current app is only at the prototype stage.

Biostatistician Reviewer

Strengths: A prospective, randomized clinical trial is the second aim of this study; the first is further development of the app based on preliminary data and the literature. Aim 2 is a randomized controlled trial (RCT) pilot test of the app. Aim 3 is basically a follow-up interview and survey of participants who used the app for the purpose of further improving it. The pilot study seems straightforward and appropriate; however, the application does not clearly lay out the hypothesis in the section labeled "hypothesis." The statistical analysis seems appropriate for repeated measures over time. The power analysis seems reasonable, and they do account for patient dropout (although 15% seems low). They do identify a qualified statistician for the project (Dr Daniel Zhao).

Weaknesses: The overall functional assessment of cancer therapy – prostate (FACT-P) score plus domain scores and others are all listed as end points; however there is no discussion of multiple comparisons. Consequently, there is a high likelihood of false positives for individual end points which may need to be confirmed in a more definitive study. This is acceptable for an exploratory pilot study but assumes that a more definitive study will be done later that includes an algorithm for handling multiplicity. The decision to restrict the study population to 3 populations of Black men could be controversial, especially if there are no apps of this type in use (other than in Sweden). This seems acceptable for a pilot study, but a more diverse population may be more informative; eg, one might expect Black men to have better outcomes if the app successfully targets their unique concerns. Conversely, it could be thought of as an enrichment strategy in that the population is selected which might benefit more from the app.

Clinical Strategy
Average Score: 7.3

Scientist Reviewer A

Strengths: This quasi-randomized clinical trial is designed appropriately to address the specific aims proposed. Four recruitment strategies are planned to ensure access to the desired patient populations of interest to the proposed study, namely, Black prostate cancer survivors. Given ongoing pilot work (DOD funded) by the PI that has demonstrated an ability to launch a pilot study within the time frame proposed, there is confidence that the proposed clinical trial will be initiated in the first year of the award.

Weaknesses: As a minor concern, the timing of a man's "survivorship" period might not be comparable across all enrollees. The ability for the study to ensure a comparable "baseline" for all prostate cancer survivors is a concern. More specifically, the efficacy of the app to improve QoL for a prostate cancer survivor who only recently completed his prostate cancer treatment might differ from a man who completed treatment more than a year ago, for instance. Although adequate alternative strategies have been detailed, there are potential challenges identified by the study team, which include the provision of smartphones during the study period to ensure steady access to the app.

Scientist Reviewer B

Strengths: The trial design is purposive sampling with quasi-random assignment of participants to standard of care either with or without the use of the SAFE-CaPS. QoL will be evaluated through a 12-month study, with participants (total of 248). The primary outcome is FACT-P. This scale is reliable and reproducible and has been validated in numerous studies.

Procurement Sensitive Information
Do not copy or distribute

Weaknesses: Participants in the app group will be provided with an Android-based smartphone and a basic cell service for the duration of the study clinical trial. If the trial is successful, the proposal does not address how they will implement an app to those without access to smartphone technology. Moreover, the inclusion of various patients with different stages of cancer which are treated dramatically differently may limit the ability to detect improvements in QoL due to confounding which is not adequately controlled for in the trial of their size. There are also weaknesses in multiple comparisons.

Personnel
Average Score: 8.5

Scientist Reviewer A

The PI, Motolani Ogunsanya, earned her PhD and MSc from the University of Texas at Austin in pharmaceutical sciences and a BPharm from the University of Lagos, Nigeria. She is currently an assistant professor in the Department of Pharmacy, Clinical and Administrative Sciences, and the Tobacco Settlement Endowment Trust (TSET) Health Promotion Research Center at University of Oklahoma Health Sciences Center (OUHSC).

Strengths: The PI is a new investigator with an established research career investigating health disparities and health outcomes in underserved minority communities. She is budgeted at 40% effort for all 3 years of the study. There is clear dedication and expertise in addressing disparities in minority populations across the study team. Other key personnel on the study include strong population science support (Dr Darla Kendzor and Dr Kathleen Dwyer) as well as strong biostatistical (Dr Yan Daniel Zhao) and community outreach and engagement support (Dr Zsolt Nagykaldi). Dr Andrew McIntosh will provide pertinent expertise in urologic oncology. Letters of support are very strong.

Weaknesses: The PI's effort at 40% seems a little high. The other key personnel are at fairly low percent effort (ranging from 2% to 5%). The Co-Is' effort could be higher, particularly Dr McIntosh's as he appears to be the only Co-I with clear urologic oncology expertise. While there is clear dedication and expertise in addressing disparities in minority populations across the study team, it is less clear how the collective knowledge base is specific to prostate cancer with the exception of Dr McIntosh and a current award that the PI is on (5% effort), examining a smartphone app to promote education around PSA screening from the Presbyterian Health Foundation.

Scientist Reviewer B

Strengths: The team has funded experience using in-depth, semistructured, qualitative interviews with 31 ethnically diverse Black prostate cancer survivors. The PI has expertise in health outcomes research among underserved minority groups using mixed methodologies.

Weaknesses: No weaknesses were noted.

Research Transition Plan
Average Score: 8.5

Scientist Reviewer A

Strengths: The transition plan is well developed and articulated and uses the Consolidated Framework for Implementation Research (CFIR) for framing. There are reasonable and appropriate milestones and deliverables proposed to advance the study results toward patient impact. It seems highly probable that

the PI will secure additional funding upon completion of the study findings, particularly to advance the next iteration of the app development into clinical practice and the patient community.

Weaknesses: The plans for distribution of the findings or intervention to the targeted disparity population(s), as well as the wider prostate cancer community, could be more developed. In particular, it is important to understand how the findings to refine the app will be expanded to include larger subsets of Black prostate cancer survivors, given the modest sample sizes upon which data will be generated.

Scientist Reviewer B

Strengths: To enhance implementation, the team plans to apply for a Small Business Innovation Research (SBIR) grant from the National Science Foundation (NSF).

Weaknesses: The assessment tool was developed by the in-house mHealth shared resource. It is unclear what the limitations of the scalability of an in-house tool will be.

UNSCORED CRITERIA

Data and Resource Sharing

Scientist Reviewer A

The data and resource sharing plan is robust and clearly detailed.

Scientist Reviewer B

The app appears to be accessible to the public.

Budget

Scientist Reviewer A

The direct costs do not exceed the allowable direct costs. The budget is appropriate for the proposed research.

Scientist Reviewer B

The budget appears appropriate.

Environment

Scientist Reviewer A

The intellectual and material property plan and statements are appropriate. The scientific environment and resources provided by the OUHSC to the study team, and in support of the proposed research, are very strong. There is clear availability of, and accessibility to, the necessary facilities and resources for the success of the proposed work. The quality and extent of organizational support are highly appropriate.

Scientist Reviewer B

The scientific environment is strong.

Procurement Sensitive Information
Do not copy or distribute

Application Presentation

Scientist Reviewer A

The writing, clarity, and presentation of the application components influenced the review only in a positive manner.

Scientist Reviewer B

This was a well presented and well articulated application.
