# Supplementary material for: A Culturally Tailored Mobile Health Intervention to Improve Quality of Life in Black Survivors With Prostate Cancer: Protocol for a Stratified Randomized Controlled Trial
Source: JMIR Res Protoc. 2026 Mar 24;15:e81503. doi: 10.2196/81503 (PMC13058526; doi:10.2196/81503)
Supplement: Multimedia Appendix 3 [file resprot_v15i1e81503_app3.docx]

**Appendix A**

**Daily Ecological Momentary Assessment Items**

**Symptom Monitoring Questions**

1. *Yesterday, I felt more body ache than usual.*
    Response Options:
     0 = Not at all
     1 = A little bit
     2 = Somewhat
     3 = Quite a bit [Alert Research Team]
     4 = Very Much [Alert Research Team]
    (Skip to Question 4 if response is "0" or "1")
2. *On a scale of 1 to 10, how would you rate the impact of the pain you are experiencing now?*
    Response: Slider scale (1–10)
3. *On a scale of 1 to 10, how would you rate the impact of pain on your social activities?*
    Response: Slider scale (1–10)
4. *In the last 24 hours, my sleep quality was…*
    Response Options:
     1 = Very Poor
     2 = Poor
     3 = Fair
     4 = Good
     5 = Very Good
    (Skip to Question 6 if response is "3", "4", or "5")
5. *Rate how much these sleep problems affect your quality of life.*
    Response Options:
     1 = A great deal
     2 = Much
     3 = Somewhat
     4 = Little
     5 = Never
6. *Yesterday, I felt more loss of energy/fatigue than usual.*
    Response Options:
     0 = Not at all
     1 = A little bit
     2 = Somewhat
     3 = Quite a bit [Alert Research Team]
     4 = Very Much [Alert Research Team]
    (Skip to Question 8 if response is "0" or "1")
7. *On a scale of 1 to 10, how would you rate the impact of the loss of energy/fatigue you are experiencing now?*
    Response: Slider scale (1–10)
8. *I have gained a significant amount of weight.*
    Response Options:
     0 = Not at all
     1 = A little bit
     2 = Somewhat
     3 = Quite a bit
     4 = Very Much
    (Skip to Question 10 if response is "0" or "1")
9. *Rate how much this weight gain affects your quality of life.*
    Response Options:
     1 = A great deal
     2 = Much
     3 = Somewhat
     4 = Little
     5 = Never
10. *Yesterday, there was blood in my urine.*
     Response Options:
      1 = Yes [Alert Research Team]
      2 = No
11. *I had difficulty urinating yesterday.*
     Response Options:
      0 = Not at all
      1 = A little bit
      2 = Somewhat
      3 = Quite a bit [Alert Research Team]
      4 = Very Much [Alert Research Team]
     (Skip to Question 13 if response is "0" or "1")
12. *Rate how much these urinary problems have affected your quality of life in the past 24 hours.*
     Response Options:
      1 = A great deal
      2 = Much
      3 = Somewhat
      4 = Little
      5 = Never
13. *I urinated more than usual yesterday.*
     Response Options:
      0 = Not at all
      1 = A little bit
      2 = Somewhat
      3 = Quite a bit
      4 = Very Much
14. *I had trouble moving my bowels yesterday.*
     Response Options:
      0 = Not at all
      1 = A little bit
      2 = Somewhat
      3 = Quite a bit
      4 = Very Much
     (Skip to Question 16 if response is "0" or "1")
15. *Rate how much loss of bowel control has affected your quality of life in the past 24 hours.*
     Response Options:
      1 = A great deal
      2 = Much
      3 = Somewhat
      4 = Little
      5 = Never
16. *I have experienced a significant reduction in my sexual functioning.*
     Response Options:
      0 = Not at all
      1 = A little bit
      2 = Somewhat
      3 = Quite a bit
      4 = Very Much
     (Skip to Question 18 if response is "0" or "1")
17. *Reduced sexual functioning affected my life yesterday.*
     Response Options:
      1 = Yes [Refer to Content Page about Reduced Sexual Functioning]
      2 = No

**Physical Activity**

1. *How many minutes of MODERATE leisure time physical activity did you engage in YESTERDAY?*
   Examples: brisk walking, bicycling, etc.
    Response Options:
     1 = 0–19 minutes
     2 = 20–39 minutes
     3 = 40–59 minutes
     4 = 60 minutes or more
2. *How many minutes of VIGOROUS leisure time physical activity did you engage in YESTERDAY?*
   Examples: running, aerobics, etc.
    Response Options:
     1 = 0–19 minutes
     2 = 20–39 minutes
     3 = 40–59 minutes
     4 = 60 minutes or more

**Dietary Behaviors**

1. *How many servings of fruits did you consume YESTERDAY?*
    Response Options:
     0 = None
     1 = 1–2 servings per day
     2 = 3–4 servings per day
     3 = 5 or more servings per day
     4 = Don’t know
2. *How many servings of vegetables did you consume YESTERDAY?*
    Response Options:
     0 = None
     1 = 1–2 servings per day
     2 = 3–4 servings per day
     3 = 5 or more servings per day
     4 = Don’t know
3. *Yesterday, how many 12 oz. sugar-sweetened beverages did you consume?*
    Response Options:
     0 = None
     1 = 1–2
     3 = 3 or more
4. *How many servings of fast food did you consume YESTERDAY?*
    Response Options:
     0 = None
     1 = 1–2
     3 = 3 or more

**Weekly Ecological Momentary Assessment Items**

**Positive and Negative Affect Schedule (PANAS)**
*Participants are asked to rate to what extent they have felt each of the following emotions during the past 7 days.*

1. Interested
2. Distressed
3. Excited
4. Upset
5. Strong
6. Guilty
7. Scared
8. Hostile
9. Enthusiastic
10. Proud
11. Irritable
12. Alert
13. Ashamed
14. Inspired
15. Nervous
16. Determined
17. Attentive
18. Jittery
19. Active
20. Afraid

Response Scale (for each):
 1 = Very slightly or not at all
 2 = A little
 3 = Moderately
 4 = Quite a bit
 5 = Extremely

**Patient Health Questionnaire-4 (PHQ-4)**
*Over the last 7 days, how often have you been bothered by the following problems?*

1. Feeling nervous, anxious, or on edge
2. Not being able to stop or control worrying
3. Little interest or pleasure in doing things
4. Feeling down, depressed, or hopeless

Response Scale:
 0 = Not at all
 1 = Several days
 2 = More than half the days
 3 = Nearly every day

**Monthly Ecological Momentary Assessment Items**

**Functional Assessment of Cancer Therapy-Prostate (FACT-P)**

*Participants are asked to consider the past 7 days when responding to the following items.*

**Physical Well-Being**
 48. I have a lack of energy
 49. I have nausea
 50. Because of my physical condition, I have trouble meeting the needs of my family
 51. I have pain
 52. I am bothered by side effects of treatment
 53. I feel ill
 54. I am forced to spend time in bed

Response Scale:
 0 = Not at all
 1 = A little bit
 2 = Somewhat
 3 = Quite a bit
 4 = Very much

**Social/Family Well-Being**
 55. I feel close to my friends
 56. I get emotional support from my family
 57. I get support from my friends
 58. My family has accepted my illness
 59. I am satisfied with family communication about my illness
 60. I feel close to my partner (or main supporter)
 61. I am satisfied with my sex life

Response Scale:
 0 = Not at all
 1 = A little bit
 2 = Somewhat
 3 = Quite a bit
 4 = Very much

**Emotional Well-Being**
 62. I feel sad
 63. I am satisfied with how I am coping with my illness
 64. I am losing hope in the fight against my illness
 65. I feel nervous
 66. I worry about dying
 67. I worry that my condition will get worse

Response Scale:
 0 = Not at all
 1 = A little bit
 2 = Somewhat
 3 = Quite a bit
 4 = Very much

**Functional Well-Being**
 68. I am able to work (including work at home)
 69. My work (including work at home) is fulfilling
 70. I am able to enjoy life
 71. I have accepted my illness
 72. I am sleeping well
 73. I am enjoying the things I usually do for fun
 74. I am content with the quality of my life right now

Response Scale:
 0 = Not at all
 1 = A little bit
 2 = Somewhat
 3 = Quite a bit
 4 = Very much
